# Analysis of key performance indicators of a 4G LTE network based on experimental data obtained from a densely populated smart city

**DOI:** 10.1016/j.dib.2020.105304

**Published:** 2020-02-17

**Authors:** Agbotiname Lucky Imoize, Kehinde Orolu, Aderemi Aaron-Anthony Atayero

**Affiliations:** a Department of Electrical and Electronics Engineering, Faculty of Engineering, University of Lagos, Lagos State, Nigeria; b Department of Systems Engineering, Faculty of Engineering, University of Lagos, Lagos State, Nigeria; c Department of Electrical and Information Engineering, Covenant University, Ota, Ogun State, Nigeria

**Keywords:** Experimental data, 4G LTE network, Key Performance indicators (KPIs), Drive Test (DT), Propagation measurements, Statistical analysis, Probability density, Smart city

## Abstract

Key performance indicator (KPI) data provide candidate information required for effective network planning, performance analysis and optimization. However, inadequate KPI data could limit efficient network planning leading to escalating operational cost, and this could adversely affect the subscribers of the network. To this end, this article presents radio frequency (RF) measurements and evaluation of KPIs taken at 1876.6MHz with a bandwidth of 10MHz, for an operational 4G LTE network in Nigeria. The measurements campaign specifically examine the behaviour of the RSRP, RSRQ, RSSI, SINR, PCC PHY DL Throughput, and the PDCP DL Throughput. Huawei Technologies Modem E392 was used for the propagation measurements, and RF measurements cover three evolved node base stations (eNodeBs) with average heights of 25 m. The geographical coordinates of the sites are as follows: Site 1 (Latitude 6.43543333; Longitude 3.44539667), Site 2 (Latitude 6.55639500; Longitude 3.36693333), and Site 3 (Latitude 6.51879500; Longitude 3.39911000). The E392 4G (LTE) Modem is capable of propagation measurements at the various LTE frequency bands, enables LTE download Speed of 100 Mbit/s, supports LTE upload Speed of 50 Mbit/s, utilizes LTE 2x2 MIMO (Multiple Input Multiple Output), and supports 64QAM (Quadrature Amplitude Modulation). The Drive Test (DT) Software version-Genex prove V16, and Genex Assistance V16 were deployed, and the test car carried a test terminal station, a GPS, a Windows supported Computer, and the accompanying drive test system. The test vehicle was driven such that it considered the actual road traffic conditions at a relatively medium speed of up to 30km/h with uniformity thereby reducing possible Doppler effects. Terminal connection was established, and data download services was started (using file transfer protocol - ftp, a drive test software, which has the function to download a large file of around 20GB). Thereafter, the download simultaneous file downloading limit was set to 5 files (such that 5 files can be downloaded simultaneously with quality download speed). When connection drops, simultaneous connection was re-established using the ftp software, and drive test was carried out within a planned cluster on a bright and sunny day. Statistical descriptions and probability distribution functions of the KPI data is reported and interdependence amongst the KPIs are presented to ease understanding of the interrelationships among the tested KPIs. The data reported would find useful applications in RF planning, radio channel measurements and modelling, feasibility studies and formulation of appropriate regulatory policies for wireless communication systems. Network operators could leverage on the data for appropriate KPI analyses, radio resources management, and research and development.

**Table:** Specifications Table

Subject	Engineering and Technology
Specific subject area	Wireless Communications Engineering
Type of data	Tables, Graphs, Charts, Figures
How data were acquired	The experimental data presented in this article were acquired through extensive drive test in and around Lagos, an emerging smart city in Nigeria. The DT equipment comprising of a test terminal station- Huawei Modem E392 (4G LTE Modem), Global Positioning System (GPS) equipment and the associated drive test system were carefully assembled in test car. The car was driven at a near constant speed of 30km/h to avoid or minimize Doppler effects, and the KPIs were measured and automatically recorded for further processing.
Data format	Raw and Analysed
Parameters for data collection	The parameters measured and tested comprise of the key performance indicators such as the Reference Signal Received Power (RSRP), Signal-to-Interference-plus-Noise Ratio (SINR), Received Signal Strength Indicator (RSSI), Reference Signal Received Quality (RSRQ), Packet Data Convergence Protocol Downlink Throughput (PDCP DL Throughput), and the Primary Component Carrier Physical Downlink Throughput (PCC PHY DL Throughput) [1].
Description of data collection	The KPI data were collected from fixed transmitters referred to as the 4G LTE base station (BS) or evolved node base station (eNodeB) with average heights of 25 m, commercial equipment belonging to one of the network operators in Nigeria. The Drive Test (DT) equipment captured the SINR, RSRP, RSRQ, RSSI, and other KPI information from the active sectors of the eNodeBs. The specifications and network design parameters were given due consideration following the manufacturers’ directives and instructions.
Data source location	The key performance indicator (KPI) data reported in this article were collected in and around three eNodeB sites with the following coordinates; Site 1 (Latitude 6.43543333; Longitude 3.44539667), Site 2 (Latitude 6.55639500; Longitude 3.36693333), and Site 3 (Latitude 6.51879500; Longitude 3.39911000), located in one of Africa's fastest growing smart city, Lagos, Nigeria.
Data accessibility	A detailed datasets on the measured KPIs taken at 1876.6MHz with a 10MHz bandwidth, of a functional 4G LTE network is provided as a supplementary file attached to this article in a spreadsheet format for easy accessibility and data reusability.

**Table:** 

**Value of the Data** • The experimental data reported in this article will enhance further research in the field of wireless communications engineering, especially in the area of radio channel measurements and key performance indicator analyses in dense urban propagation environments [[2], [3], [4]]. • The data will also be of immense benefits to: 1) Radio Network Engineers for assessing and determining the optimal location of base stations (BSs), radio channels and radio coverage estimations, and capacity improvements. 2) Radio Frequency Planning Engineers for radio frequency planning, frequency assignments and network optimization, drive testing and optimal allocation of radio resources, and quality of service (QoS) analyses. 3) Regulatory and Compliance Engineers can also leverage on the data to provide suitable KPI benchmarks for mobile network operators [[5], [6], [7]]. • The KPI data will provide further insights and development of experiments in the area of radio network design, development and validation of high precision propagation models for accurate prediction of pathloss in environments where radio signals are severely impacted by multi-scattering attenuation under different environmental conditions [7,8]. • The data could also find additional use as candidate materials for class room studies (testing and validating theoretical and simulation results) [[9], [10], [11]].

## 1 Data

Wireless communication data provide useful information pertinent to the development of communication equipment, standards and specifications, conducting high-level feasibility studies during initial deployment of telecommunication infrastructure, and providing accurate evaluation of the quality of service (QoS) [12] in order to enhance the quality of user experience (QoE) [13]. Generally, wireless communication systems are designed to transfer data from a source to a destination (from the transmitter to the receiver). As wireless systems continue to grow and evolve to accommodate upward scaling traffic requirements following the rapid deployment of 4G LTE networks and the evolving 5G and beyond wireless systems [2,5,14], analysis of the key performance indicators increasingly becomes a concern. Toward this end, the need to critically examine and evaluate the KPIs of an operational 4G LTE network becomes imperative. This is considered highly important due to the enormous benefits such data provide; useful information about the performance of the network in real time, and present a suitable platform to furnish improvement initiatives on the existing network structure in terms of coverage and capacity [15,16]. Finally, the data could aid the development of advanced modulation techniques [[17], [18], [19]], and foster the development of energy efficient wireless communications systems [[20], [21], [22]].

In this article, analysis of some selected KPIs of an operational 4G LTE network is presented. The tested KPIs include the RSRP, RSRQ, RSSI, SINR, PCC PHY DL Throughput, and the PDCP DL Throughput. These KPIs were measured at a 4G LTE frequency of 1876.6MHz with 10MHz bandwidth. The extensive RF measurements span a propagation distance of up to 2km, and measured KPIs were extracted and analysed in IBM SPSS Statistics and MATLAB.

**Fig. 1:**
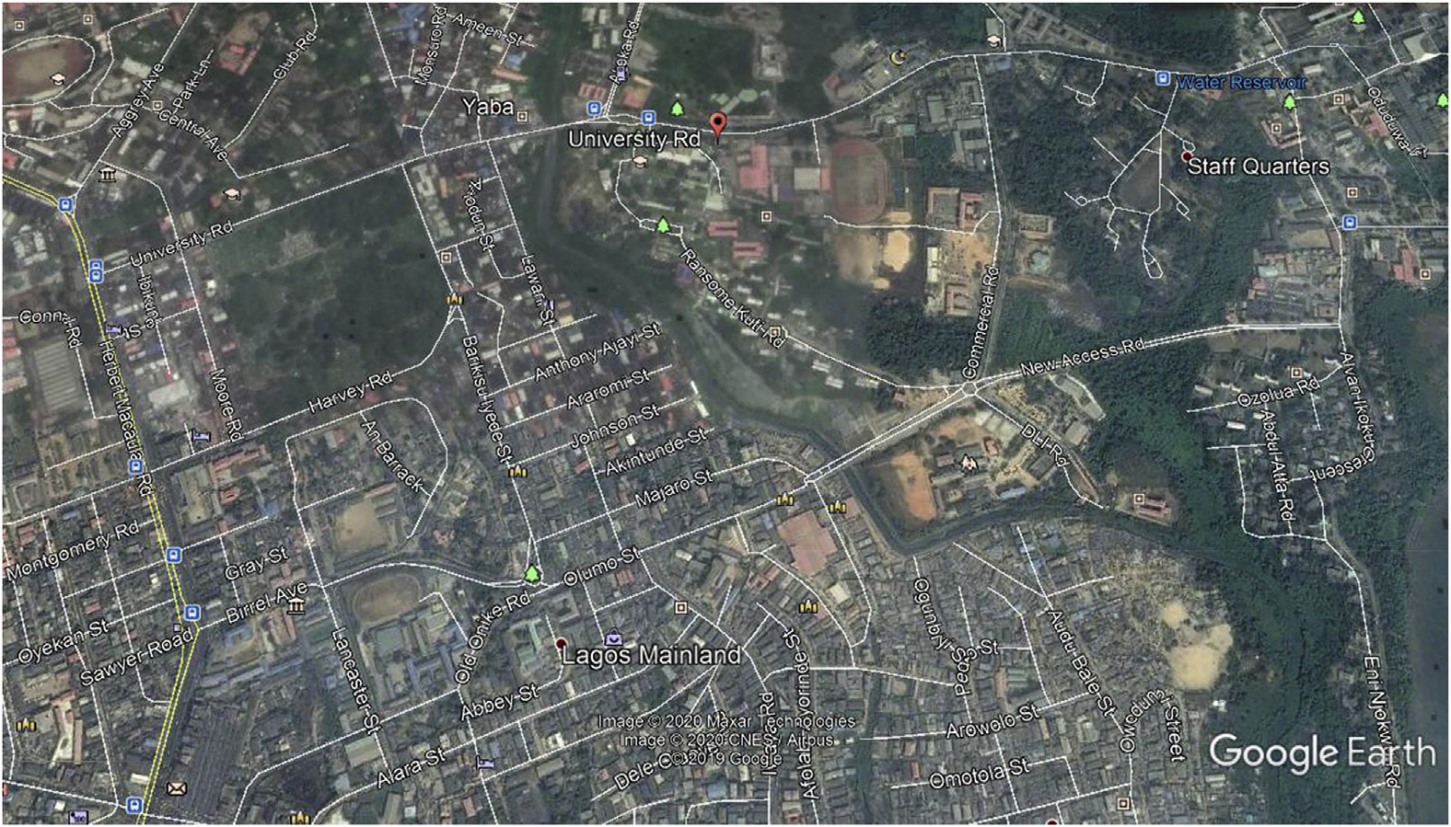
Aerial view of the measurements environment.

**Fig. 2:**
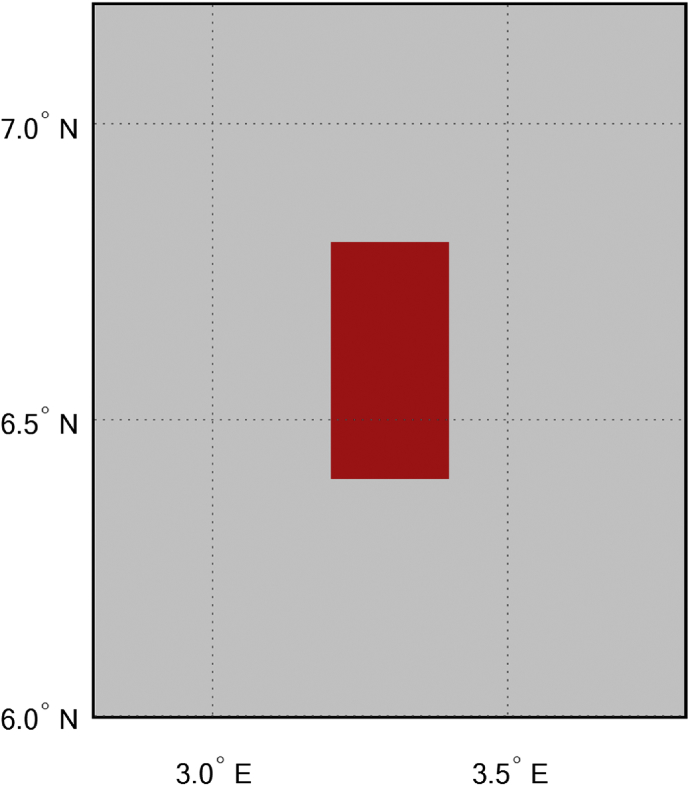
Geographical coordinates of the measurements environment.

**Fig. 3:**
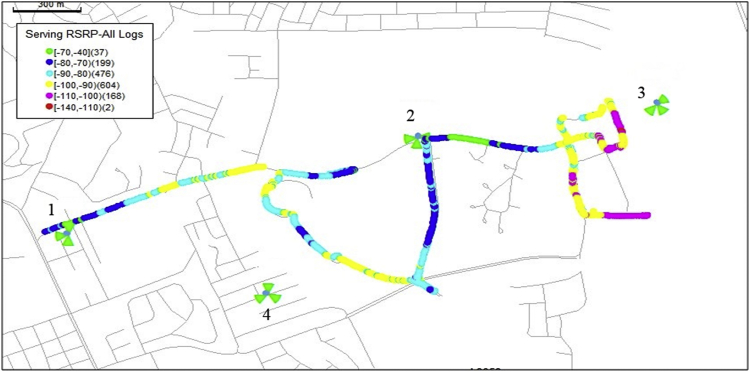
Trajectories of 4G LTE RSRP performance distribution.

**Fig. 4:**
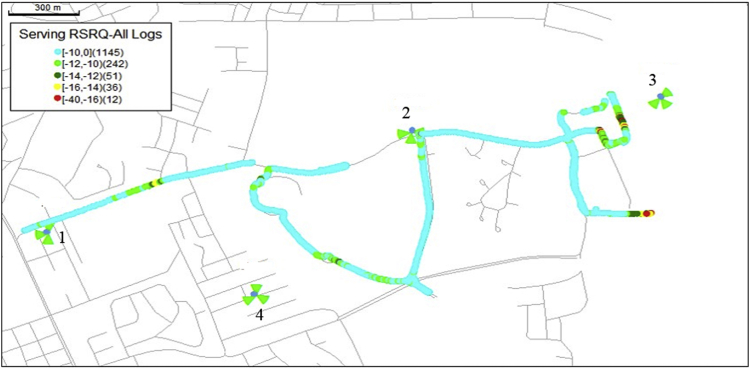
Trajectories of 4G LTE RSRQ performance distribution.

**Fig. 5:**
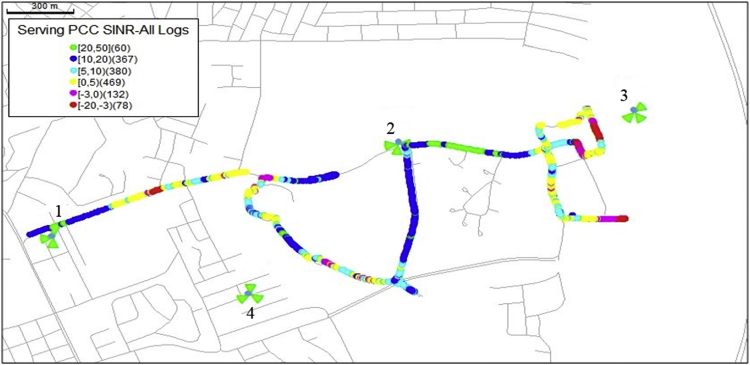
Trajectories of 4G LTE SINR performance distribution.

**Fig. 6:**
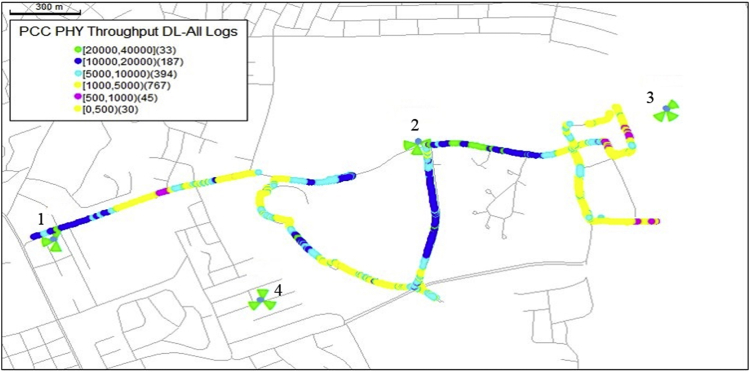
Trajectories of 4G LTE PCC PHY Throughput performance distribution.

**Fig. 7:**
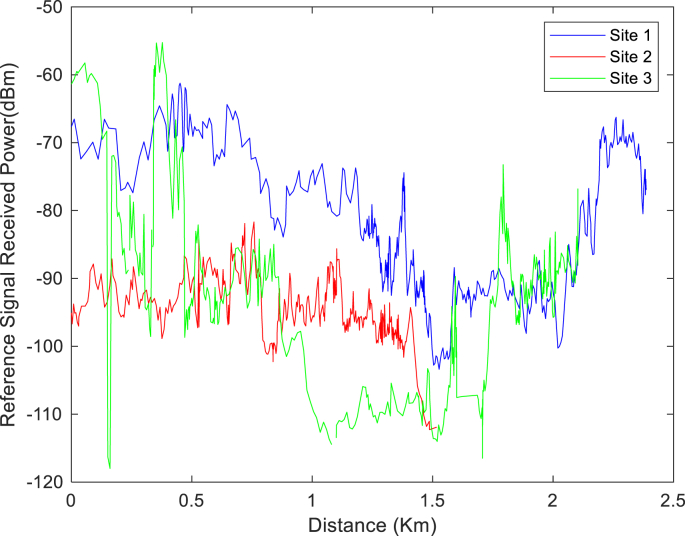
Measurements of RSRP at sites 1–3.

**Fig. 8:**
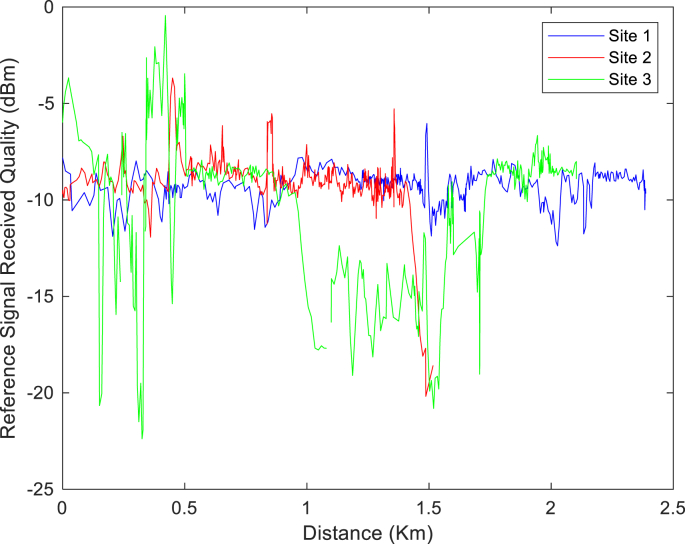
Measurements of RSRQ at sites 1–3.

**Fig. 9:**
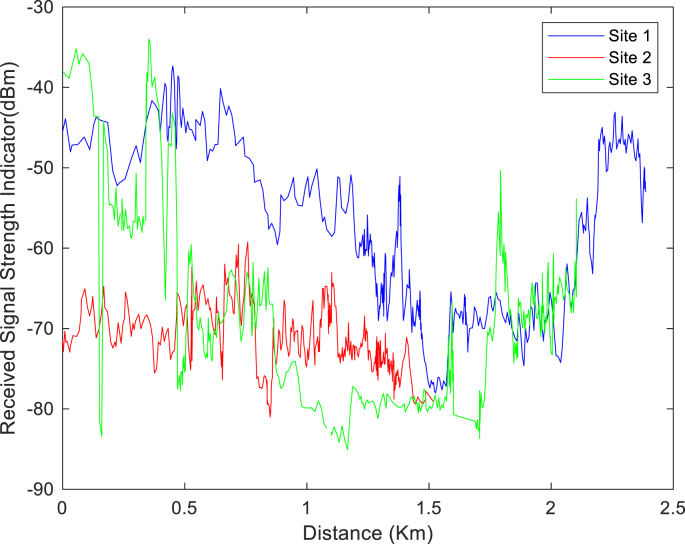
Measurements of RSSI at sites 1–3.

**Fig. 10:**
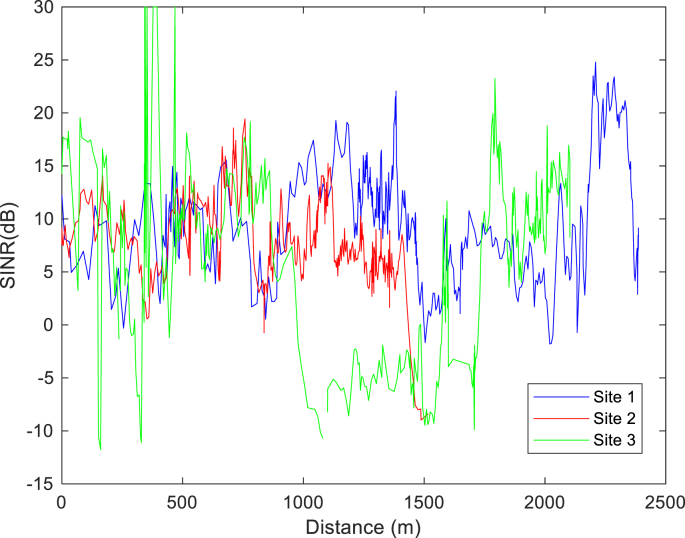
Measurements of SINR at sites 1–3.

**Fig. 11:**
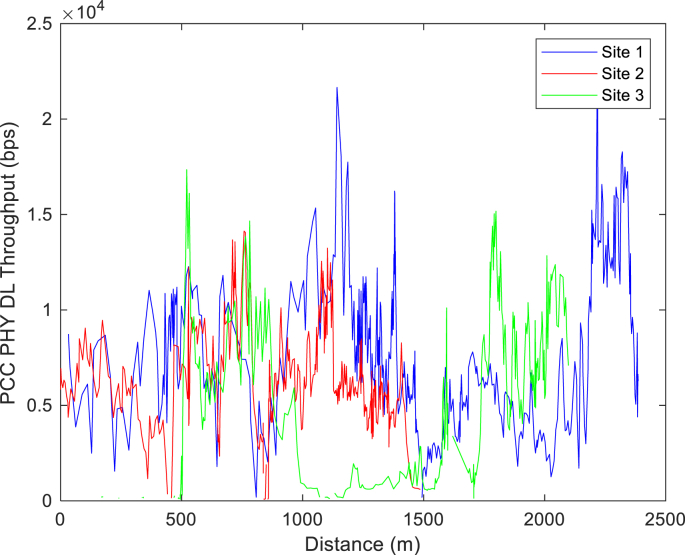
Measurements of PCC PHY DL Throughput at sites 1–3.

**Fig. 12:**
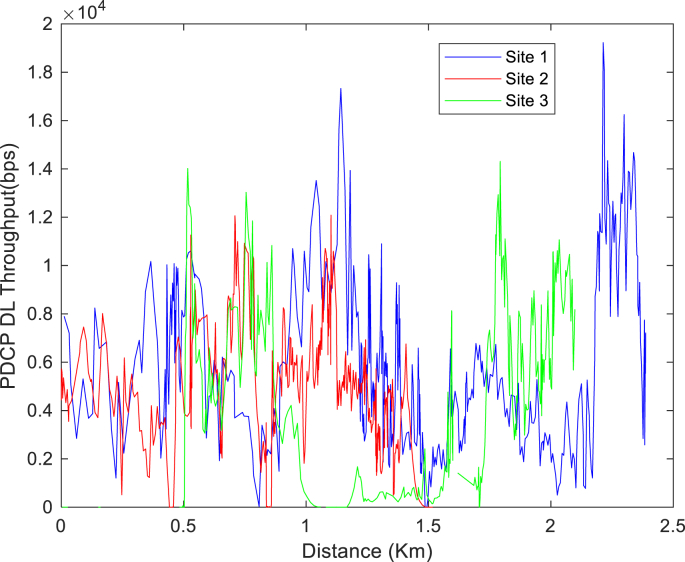
Measurements of PDCP DL Throughput at sites 1–3.

The KPIs derived from the experimental data are briefly described as follows. First, the aerial view and the geographical coordinates of the measurements environment are as shown in Fig. 1, Fig. 2, respectively. The trajectories of 4G LTE RSRP, RSRQ, SINR, and PCC PHY Throughput performance distributions are as shown in Fig. 3, Fig. 4, Fig. 5, Fig. 6, respectively. The specific KPI information are presented in Fig. 7, Fig. 8, Fig. 9, Fig. 10, Fig. 11, Fig. 12. Specifically, the RSRP measured at Sites 1–3 is given in Fig. 7, and Fig. 8 shows the RSRQ measured at Sites 1–3. Fig. 9 represents the RSSI measured at Sites 1–3, Fig. 10 gives the SINR measured at Sites 1–3, and Fig. 11 presents the PCC PHY DL Throughput measured at Sites 1–3. Finally, Fig. 12 shows the PDCP DL Throughput measured at Sites 1–3.

The statistics of the measured KPIs are given in Table 1, Table 2, Table 3, Table 4, Table 5, Table 6, Table 7, Table 8, Table 9. More specifically, Table 1 presents statistics of measured RSRP, RSRQ, and the RSSI at Site 1. Table 2 gives the statistics of measured SINR, PCC PHY DL Throughput, and the PDCP DL Throughput at Site 1. Table 3 represents the statistics of measured RSRP, RSRRQ, and the RSSI at Site 2. Table 4 depicts the statistics of measured SINR, PCC PHY DL Throughput, and the PDCP DL Throughput at Site 2. In addition, Table 5 presents the statistics of measured RSRP, RSRQ, and the RSSI at Site 3, whereas, Table 6 gives the statistical analysis of the measured SINR, PCC PHY DL Throughput, and the PDCP DL Throughput at Site 3. Furthermore, Table 7 gives a comparison of the measured RSRP and the RSRQ at Sites 1–3, and a comparison of the measured RSSI and the SINR at Sites 1–3 is given in Table 8. Last, Table 9 shows a comparison of the measured PCC PHY DL Throughput, and the PDCP DL Throughput at Sites 1–3.

**Table 1:** Statistical description of measured RSRP, RSRQ, and RSSI at site 1.

Statistics	RSRP (dBm)	RSRQ (dBm)	RSSI (dBm)
N	428.000	428.000	428.000
Range	42.140	6.300	40.680
Minimum	−103.380	−12.400	−78.010
Maximum	−61.240	−6.000	−37.330
Mean	−82.508	−9.247	−59.056
Std. Deviation	10.308	.768	10.158
Variance	106.257	.589	103.194
Skewness	.130	−.912	.182
Kurtosis	−1.083	2.706	−1.127

**Table 2:** Statistical description of measured PCC SINR, PCC PHY DL Throughput, and PDCP DL Throughput at site 1.

Statistics	PCC SINR (dB)	PCC PHY DL Throughput (bps)	PDCP DL Throughput (bps)
N	428.000	428.000	428.000
Range	26.590	23885.700	19223.970
Minimum	−1.800	.000	.000
Maximum	24.790	23885.700	19223.970
Mean	10.085	7557.274	5653.074
Std. Deviation	5.545	3888.595	3423.003
Variance	30.749	15121167.752	11716951.633
Skewness	.364	.721	.978
Kurtosis	−.318	.672	.804

**Table 3:** Statistical description of measured RSRP, RSRQ, and RSSI at site 2.

Statistics	RSRP (dBm)	RSRQ (dBm)	RSSI (dBm)
N	523.000	523.000	523.000
Range	30.590	16.500	21.790
Minimum	−112.270	−20.180	−81.020
Maximum	−81.680	−3.680	−59.230
Mean	−94.810	−9.069	−71.625
Std. Deviation	4.315	1.356	3.845
Variance	18.619	1.839	14.783
Skewness	.107	.107	.107
Kurtosis	.213	.213	.213

**Table 4:** Statistical description of measured SINR, PCC PHY DL Throughput, and PDCP DL Throughput at site 2.

Statistics	PCC SINR (dB)	PCC PHY DL Throughput (bps)	PDCP DL Throughput (bps)
N	523.000	523.000	523.000
Range	28.420	14129.570	12084.160
Minimum	−8.980	.000	.000
Maximum	19.440	14129.570	12084.160
Mean	7.456	6031.110	4825.821
Std. Deviation	3.838	2583.239	2488.209
Variance	14.734	6673125.510	6191182.572
Skewness	.107	.107	.107
Kurtosis	.213	.213	.213

**Table 5:** Statistics of measured RSRP, RSRQ, and RSSI at site 3.

Statistics	RSRP (dBm)	RSRQ (dBm)	RSSI (dBm)
N	411.000	411.000	411.000
Range	62.740	21.940	51.050
Minimum	−117.990	−22.380	−85.050
Maximum	−55.250	−.440	−34.000
Mean	−93.444	−10.412	−68.474
Std. Deviation	13.701	3.926	11.636
Variance	187.706	15.412	135.403
Skewness	.727	−.728	1.110
Kurtosis	.466	.217	.771

**Table 6:** Statistical description of measured SINR, PCC PHY DL Throughput, and PDCP DL Throughput at site 3.

Statistics	PCC SINR (dB)	PCC PHY DL Throughput (bps)	PDCP DL Throughput (bps)
N	411.000	415.000	414.000
Range	41.760	17347.690	14309.480
Minimum	−11.760	.000	.000
Maximum	30.000	17347.690	14309.480
Mean	5.905	4309.303	3568.004
Std. Deviation	9.157	4244.998	3746.405
Variance	83.842	18020006.824	14035549.722
Skewness	.072	.656	.706
Kurtosis	−.321	−.690	−.671

**Table 7:** Comparison of the statistics of measured RSRP and RSRQ at sites 1–3.

Statistics	RSRP (dBm)			RSRQ (dBm)		
	SITE 1	SITE 2	SITE 3	SITE 1	SITE 2	SITE 3
N	428.000	523.000	411.000	428.000	523.000	411.000
Mean	−82.508	−94.810	−93.444	−9.247	−9.069	−10.413
Std. Deviation	10.308	4.315	13.701	0.768	1.356	3.926
Variance	106.257	18.619	187.706	0.589	1.839	15.412
Skewness	0.130	−0.025	0.727	−0.912	−2.768	−0.728
Std. Error of Skewness	0.118	0.107	0.120	0.118	0.107	0.120
Kurtosis	−1.083	1.677	0.466	2.706	22.998	0.217
Std. Error of Kurtosis	0.235	0.213	0.240	0.235	0.213	0.240
Range	42.140	30.590	62.740	6.340	16.500	21.940
Minimum	−103.380	−112.270	−117.990	−12.380	−20.180	−22.380
Maximum	−61.240	−81.680	−55.250	−6.040	−3.680	−0.440

**Table 8:** Comparison of the statistics of measured RSSI and SINR at sites 1–3.

Statistics	RSSI (dBm)			SINR (dB)		
	SITE 1	SITE 2	SITE 3	SITE 1	SITE 2	SITE 3
N	428.000	523.000	411.000	428.000	523.000	411.000
Mean	−59.056	−71.625	−68.474	10.086	7.456	5.905
Std. Deviation	10.158	3.845	11.636	5.545	3.838	9.157
Variance	103.194	14.783	135.403	30.749	14.734	83.842
Skewness	0.182	0.456	1.110	0.364	−0.129	0.072
Std. Error of Skewness	0.118	0.107	0.120	0.118	0.107	0.120
Kurtosis	−1.127	0.111	0.771	−0.318	2.190	−0.321
Std. Error of Kurtosis	0.235	0.213	0.240	0.235	0.213	0.240
Range	40.680	21.790	51.050	26.590	28.420	41.760
Minimum	−78.010	−81.020	−85.050	−1.800	−8.980	−11.760
Maximum	−37.330	−59.230	−34.000	24.790	19.440	30.000

**Table 9:** Comparison of the statistics of measured PCC PHY DL Throughput and PDCP DL Throughput at sites 1–3.

Statistics	PCC PHY DL Throughput (bps)			PDCP DL Throughput (bps)		
	SITE 1	SITE 2	SITE 3	SITE 1	SITE 2	SITE 3
N	428.000	523.000	415.000	428.000	523.000	414.000
Mean	7557.274	6031.110	4309.303	5653.074	4825.821	3568.004
Std. Deviation	3888.595	2583.239	4244.998	3423.003	2488.209	3746.405
Variance	15121167.752	6673125.510	18020006.824	11716951.633	6191182.572	14035549.722
Skewness	0.721	0.324	0.656	0.978	0.428	0.706
Std. Error of Skewness	0.118	0.107	0.120	0.118	0.107	0.120
Kurtosis	0.672	0.811	−0.690	0.804	0.212	−0.671
Std. Error of Kurtosis	0.235	0.213	0.239	0.235	0.213	0.239
Range	23885.700	14129.570	17347.690	19223.970	12084.160	14309.480
Minimum	0.000	0.000	0.000	0.000	0.000	0.000
Maximum	23885.700	14129.570	17347.690	19223.970	12084.160	14309.480

**Fig. 13:**
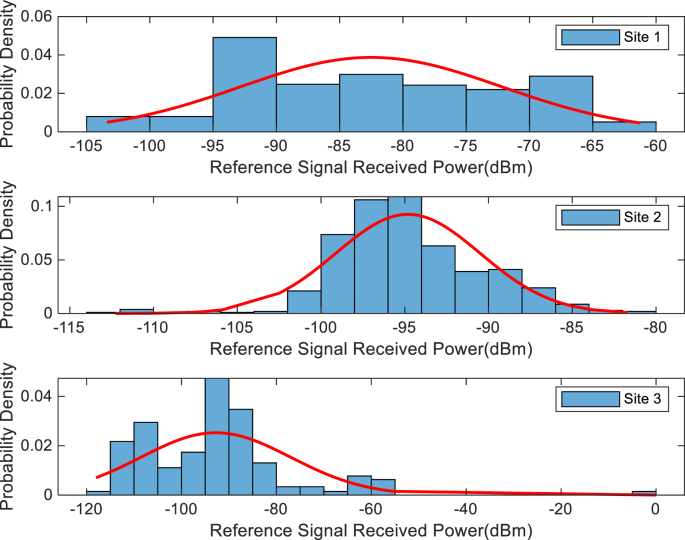
Probability density of the measured RSRP at sites 1–3.

**Fig. 14:**
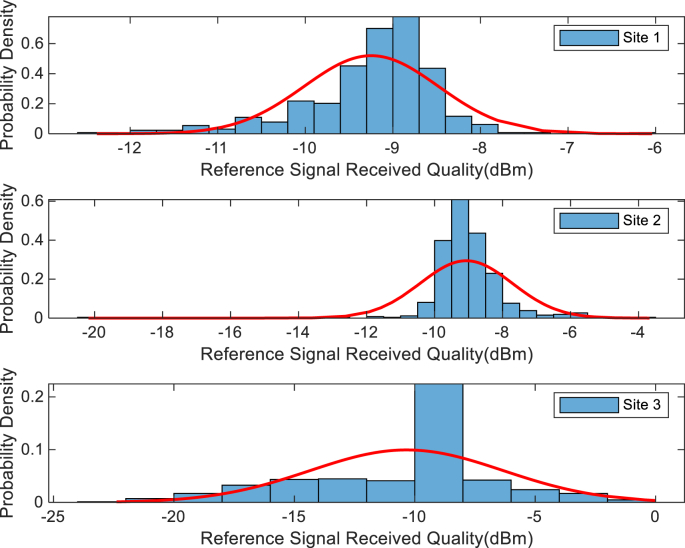
Statistics showing probability density of the measured RSRQ at sites 1–3.

**Fig. 15:**
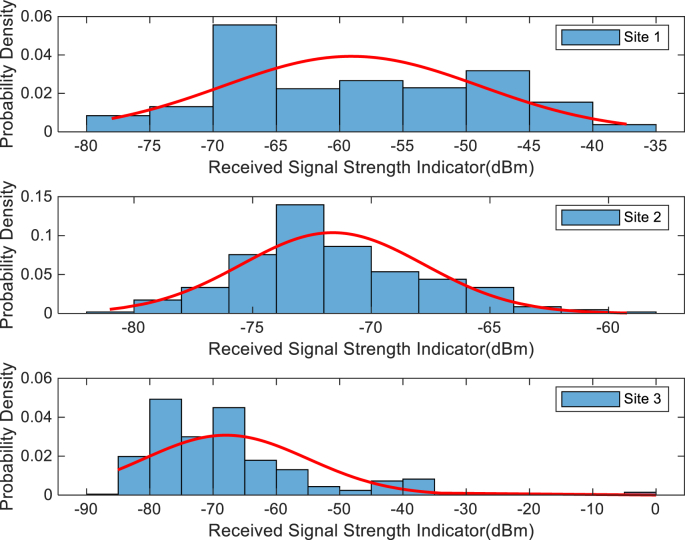
Statistics showing probability density of the measured RSSI at sites 1–3.

**Fig. 16:**
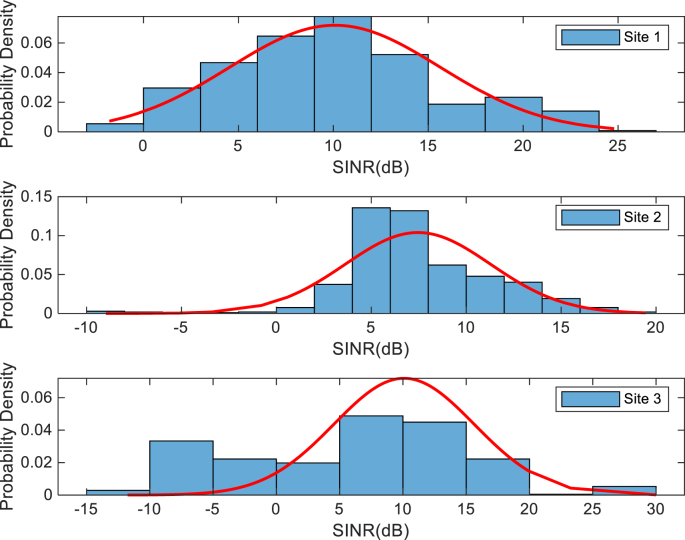
Statistics showing probability density of the measured SINR at sites 1–3.

**Fig. 17:**
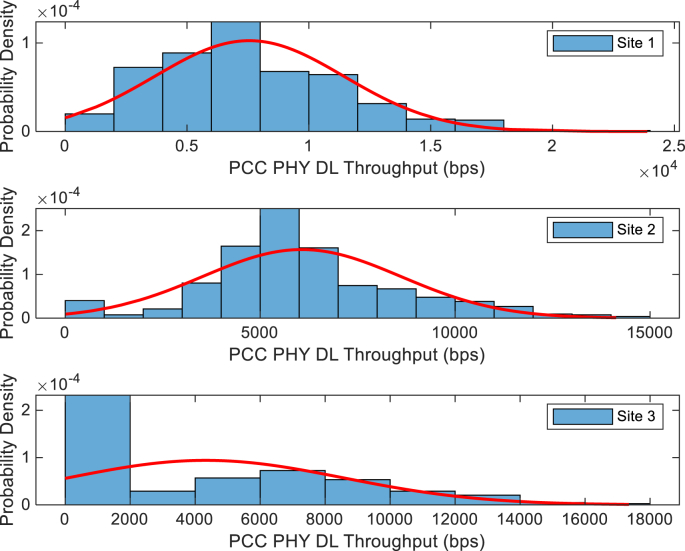
Statistics showing probability density of the measured PCC PHY DL Throughput at sites 1–3.

**Fig. 18:**
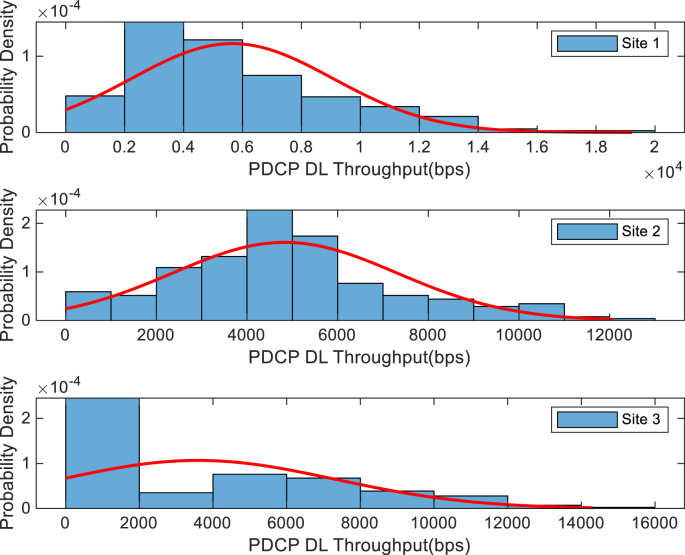
Statistics showing probability density of the measured PDCP DL Throughput at sites 1–3.

The probability distribution of the KPIs observed are given in Fig. 13, Fig. 14, Fig. 15, Fig. 16, Fig. 17, Fig. 18. Notably, Fig. 13 illustrates the probability density of the measured RSRP at Sites 1–3. Fig. 14 gives the probability density of the measured RSRQ at Sites 1–3, and Fig. 15 provides the probability density of the measured RSSI at Sites 1–3. In the same vein, Fig. 16 reports the probability density of the measured SINR at Sites 1–3, whereas, Fig. 17 represents the probability density of the measured PCC PHY DL Throughput at Sites 1–3. Finally, Fig. 18 presents the probability density of the measured PDCP DL Throughput at Sites 1–3.

## 2 Experimental design, materials, and methods

The equipment used for measurements is the Huawei Modem E392. The E392 4G (LTE) Modem offers flexibility in RF measurements and post processing of measurements data. The equipment can be used for propagation measurements at various LTE frequency bands, and supports a LTE download Speed of 100 Mbit/s, while the LTE upload Speed supported is up to 50 Mbit/s. Furthermore, the device supports LTE 2x2 MIMO and 64QAM (Quadrature Amplitude Modulation). The Drive Test (DT) Software version-Genex prove V16, and Genex Assistance V16 were selected and carefully connected and assembled in the DT car for seamless propagation measurements. The drive test car carried the test terminal station, the GPS equipment, and a personal computer (PC), and the associated drive test system.

In order to achieve quality results, the test vehicle was driven such that it considered the actual road traffic conditions at medium speed of up 30km/h with uniformity. This helps to reduce the possible impacts of Doppler effects. Afterwards, the terminal connection was established, and data download services started using file transfer protocol - ftp, a drive test software, which has the function to download a large file around or up to 20 giga bytes (GB). Thereafter, the download simultaneous file downloading limit was set to 5 files (such that 5 files can be downloaded simultaneously without significant computational cost especially on the baseband processing unit). When connection drops, simultaneous connection was re-established using the ftp software, and drive test was carried out within a planned cluster located in the geographical coordinates of the measurements environment. For data post processing, MATLAB 2018a, a product of Mathworks Incorporated, and the IBM Statistical tool (SPSS) version 24 were used.
